# Social anxiety disorder and emotion regulation problems in adolescents

**DOI:** 10.1186/s13034-019-0297-9

**Published:** 2019-09-30

**Authors:** Petra Sackl-Pammer, Rebecca Jahn, Zeliha Özlü-Erkilic, Eva Pollak, Susanne Ohmann, Julia Schwarzenberg, Paul Plener, Türkan Akkaya-Kalayci

**Affiliations:** 1 Department of Child and Adolescent Psychiatry, Medical University of Vienna, Währinger Gürtel 18-20, 1090 Vienna, Austria; 2 Department for Psychiatry and Psychotherapy, Clinical Division of Social Psychiatry, Medical University of Vienna, Währinger Gürtel 18-20, 1090 Vienna, Austria; 3 Outpatient Clinic of Transcultural Psychiatry and Migration Induced Disorders in Childhood and Adolescence, Department of Child and Adolescent Psychiatry, Medical University of Vienna, Währinger Gürtel 18-20, 1090 Vienna, Austria

**Keywords:** Social anxiety disorder (SAD), Emotion regulation, Maladaptive emotion regulation, Adaptive emotion regulation, Adolescents, Psychotherapy (cognitive behavioral therapy-CBT)

## Abstract

**Background:**

Social anxiety disorder (SAD) in adolescents may be associated with the use of maladaptive emotion regulation (ER) strategies. The present study examined the use of maladaptive and adaptive ER strategies in adolescents with SAD.

**Methods:**

30 adolescents with SAD (CLIN) and 36 healthy adolescents for the control group (CON) aged between 11 and 16 years were assessed with the standardized questionnaires PHOKI (*Phobiefragebogen für Kinder und Jugendliche*) for self-reported fears as well as FEEL-KJ (*Fragebogen zur Erhebung der Emotionsregulation bei Kindern und Jugendlichen*) for different emotion regulation strategies.

**Results:**

Compared to controls, adolescents with SAD used adaptive ER strategies significantly less often, but made use of maladaptive ER strategies significantly more often. There was a significant positive correlation between maladaptive ER and social anxiety in adolescents. Examining group differences of single ER strategy use, the CLIN and CON differed significantly in the use of the adaptive ER strategy *reappraisal* with CLIN reporting less use of *reappraisal* than CON. Group differences regarding the maladaptive ER strategies *withdrawal* and *rumination*, as well as the adaptive ER strategy *problem*-*solving* were found present, with CLIN reporting more use of *withdrawal* and *rumination* and less use of *problem*-*solving* than CON.

**Conclusions:**

Promoting adaptive emotion regulation should be a central component of psychotherapy (cognitive behavioral therapy-CBT) for social anxiety in adolescents from the beginning of the therapy process. These findings provide rationale for special therapy programs concentrating on the establishment of different adaptive ER strategies (including *reappraisal*). As an increased use of maladaptive ER may be associated with SAD in adolescents, it may be paramount to focus on reduction of maladaptive ER (for example *withdrawal* and *rumination*) from the beginning of the psychotherapy process. Incorporating more ER components into psychotherapy (CBT) could increase the treatment efficacy. Further investigations of the patterns of emotion regulation in specific anxiety groups like SAD in adolescents is needed to continue to optimize the psychotherapy (CBT) concept.

## Background

According to the Diagnostic Statistical Manual of Mental Disorders (DSM-5; American Psychiatric Association) [1], social anxiety is defined as an excessive, irrational fear and avoidance of social or performance situations due to the expectation that others will scrutinize one’s actions. Social anxiety disorder (SAD) is one of the most frequent mental health disorders [2]. Typically, it begins in childhood or adolescence [3, 4]. The average age of onset for SAD is early to mid-adolescence (median 15), but it can occur in much younger children as well [5]. SAD has a high comorbidity with other mental disorders (50–80%), particularly with other anxiety and affective disorders [6]. When left untreated, SAD runs a chronic course [7], furthermore high social anxiety can be associated with significant psychosocial impairments and reduced quality of life [8–10].

Various studies have reported that individuals with SAD have maladaptive systematic distortions in information processing [11–13] and various emotional deficits to be associated with SAD. Affected individuals showed higher intensities of negative emotions [14, 15], less emotion knowledge [16], and impaired emotion recognition [17]. Moreover, deficits in attention, interpretation and judgment or expectation were reported in individuals with SAD [11–13]. Although individuals with SAD wish to engage in social interactions, they are simultaneously overburdened by social standards. The fear of behaving inadequately in a given situation increases their social anxiety and leads to an increase in self-concentration [18–22]. Hence children with SAD quite often suffer from serious impairments in their social [23] and academic [23, 24] lives. For example, they score higher on a loneliness-scale and report having fewer friends than their age-matched peers [23]. They often dislike school and consequently attend school irregularly, or drop out entirely [23, 24]. Furthermore, SAD is strongly associated with other mental disorders [25, 26]. A comorbidity rate of up to 60% has been reported [27, 28], with the most common comorbidities being other anxiety disorders [3, 29] and affective disorders, especially depression [25, 28–31]. In a 10-year longitudinal study [32], half of the participants with SAD suffered from a depressive episode. In addition, SAD has been found to be a risk factor for alcohol and cannabis dependency [33].

Despite the fact that SAD can be very persistent [3, 28, 34] it can take years—even decades—until those suffering from SAD receive appropriate treatment [35]. There are several reasons for this. For example, only a small percentage of those affected seek professional help [3]. In addition, SAD often goes unnoticed and is therefore underdiagnosed, even by professionals [31, 36]. Furthermore, CBT (cognitive behavior therapy), which shows the strongest evidence for treating childhood SAD [37], has a success rate of 70% [38]. Maladaptive emotion regulation is suspected to play an important role in the treatment outcome of SAD especially when regarding non-responders of conventional CBT programs.

### Emotion regulation

Emotion regulation (ER) has been a booming area of research for the last 20 years, with an exponential growth in the number of related publications [39–42]. ER is defined as a person’s efforts to influence the quality, intensity, timing, expression and dynamic features of their positive and negative emotions [43, 44]. Emotion dysregulation can be defined as a state in which one’s attempts to regulate emotions fail to achieve emotion-related goals despite one’s best efforts [45], which is associated with psychopathology [46].

Emotion regulation capacities develop from childhood to adolescence to adulthood. Studies of developing individuals suggest the limited efficacy of internal regulatory strategies in early adolescence, changing to more use of adaptive strategies and decreased use of maladaptive strategies with age [47].

Emotion regulation is also discussed as a mediating variable between a risk factor (e.g., early life adversity) and the development of psychopathology.

The process-model of Gross [48] is by far the most often cited model in the field of ER [49]. It states that ER strategies can be grouped by their temporal occurrence in the ER process into either antecedent-focused or response-focused strategies [48]. In many subsequent studies, antecedent-focused strategies, like reappraisal, have proven to be superior to response-focused strategies, like suppression, in down-regulating negative emotions as well as their accompanying somatic responses [48–51]. The association between the use of different ER strategies and social, psychological, and physical wellbeing has also been investigated. The use of reappraisal resulted in less depressive symptoms, more optimism, more self-consciousness, and higher quality of life [50], as well as a favorable profile regarding the social life of participants [50, 52]. In contrast, the use of suppression showed opposite results [50, 52]. Use of the ER strategy rumination also had unfavorable results [53–55]. Ray et al. demonstrated that participants using rumination as a regulation strategy felt the emotion of anger longer and showed higher levels of activity in the central and peripheral sympathetic nervous system than those who did not use rumination [54].

Self-reported analyses data consistently identifies associations between emotion regulation abilities and symptoms of anxiety and depression in adolescents. Higher levels of rumination were associated with greater symptoms of social anxiety [56]. This was recently confirmed in a meta-analysis of 35 studies in adolescents (aged between 13 and 18 years), demonstrating that compared to healthy individuals, those with anxiety and depressive disorders engaged in less reappraisal, problem solving, and acceptance (adaptive regulatory strategies) and more avoidance, suppression and rumination (maladaptive strategies) [41].

There is very little data about potential ER deficits in children and adolescents with SAD. The first evidence comes from a study published by Lange and Tröster [57], which found that children and adolescents with SAD used maladaptive ER strategies significantly more often and adaptive ER strategies significantly less often than healthy controls. The study from Young et al. [58] instigated the role of ER in adolescents and suggested that increased use of maladaptive ER strategies may mediate the association between adversity and psychopathology.

As an increased use of maladaptive ER may be associated with SAD in children and adolescents, it may be helpful to include the reduction of maladaptive ER to establish adaptive ER at the beginning of psychotherapeutic treatment strategies as one of the most important focuses in the psychotherapy. Self-esteem is positively influenced by having good ER strategies, which make the treatment of SAD more successful.

## Aims of the study

In the current study, the emotion regulation of adolescents diagnosed with SAD (CLIN) was investigated and compared with a healthy control group (CON). Based on existing data, it was assumed that adolescents with SAD would use adaptive ER strategies less often and maladaptive ER strategies more often than CON. In addition, the ability *of certain ER strategies to predict the membership of participants to the* CLIN and CON *was explored.*

## Methods

### Study design and participants

The present study is a case–control study aimed to compare emotion regulation of adolescents suffering from SAD (CLIN) and healthy controls (CON).

CLIN consisted of 30 adolescents (in- and out-patient) seeking treatment at the Department of Child and Adolescent Psychiatry at the Medical University Vienna. All fulfilled the ICD-10 diagnostic criteria for SAD based on two independent raters with ample clinical experience using ICD-10 criteria. Thirty-six healthy age-matched adolescents without any psychiatric disorders served as controls. Additionally, at least one parent of each participant took part in the study. Participants of both groups were aged between 11 and 16 years.

Participants of CON were recruited at youth clubs in Vienna after getting their parents’ consent. To insure that adolescents of CON were psychologically healthy they were screened with the PHOKI (*Phobiefragebogen für Kinder und Jugendliche*) [59] and the Youth Self-Report (YSR) [60]. Parents completed the Child Behavior Checklist 4-18 (CBCL/4-18) [61]. In addition a psychiatric exploration was performed to confirm the absence of any mental health disorders or severe medical conditions.

The same two independent raters with ample clinical experience did the assessment for the present study in the CLIN as well as CON. Participants of the CLIN completed the questionnaires at the clinic, testing of CON was conducted at their place of recruitment.

Exclusion criteria for both groups were: (a) an IQ below 70, and (b) insufficient knowledge of the German language. As some of the used questionnaires for the study were available only in German, adolescents with insufficient German language skills were not involved in the study. The data for the present study was collected over a 2-year period. Additional exclusion criteria for CON was a history of a mental health disorder or any psychiatric/psychological/psychotherapeutic treatment in the present or past.

In the present study the gender distribution was unequal, as more male patients with the diagnosis of social phobia (according to ICD-10 criteria) were admitted to our clinic during the study period, and fewer female patients compared to male patients could participate in the study. The control group was recruited from youth clubs in Vienna. More females decided for voluntary participation compared to males. Because of this mismatch between male and female participant numbers, participants are matched by age but not by sex. As the number of the study sample was small, gender-matching could not be done. In the CLIN as well as CON, the same assessment process for recruitment and selection was conducted.

### Measures

To ensure comparability between CLIN and CON, various demographic variables were collected, including age of parents, highest parental level of education, family status (parents living together/parents are separated), number of siblings, and housing conditions.

Various self-reported fears, such as school phobia, separation anxiety, or social anxiety, were assessed using the standardized questionnaire, PHOKI (*Phobiefragebogen für Kinder und Jugendliche*) [59]. SAD was diagnosed by two experts (psychologist and psychiatrist) and both confirmed diagnosis of SAD with the help of ICD-10 (ICD-10 classification of mental and behavioural disorders) [62]. PHOKI [59] was used for more detailed information about SAD and other anxiety symptoms.

The internal consistencies, which lie between α = .70 and α = .93 for the subscales and the total scale, are given as a measure of the reliability.

The control group was recruited from a group of scouts by word of mouth, who to date had no psychological symptoms diagnosed and had no psychiatric/psychological/psychotherapeutic treatment and had undetectable values by Youth Self-Report (YSR) [60] assessment.

The Child Behavior Checklist 4-18 (CBCL/4-18) [61] was used to get a parents’ rating of symptom presence and severity. CBCL/4-18 is a paper and pencil instrument, in which parents assess the mental health of their children concerning three aspects: overall diseases, internal and external problems. The CBCL/4-18 as well as YSR [60] consists of 8 scales (Withdrawn, Somatic complaints, Anxious/depressed, Social problems, Thought problems, Attention problems, Delinquent behaviour and Aggressive behaviour) which assess the mental health of the children and adolescents. At least one parent of each participant completed the (CBCL/4-18) [61], which assesses internalizing and externalizing emotional and behavioral problems in children. The instrument is considered to be a general indicator of mental health problems in youth. The CBCL/4-18 has a high reliability above α = .80, and the internal consistency is about α = .80 [61].

The CBCL/4-18 [61] cut-off score is above 70 (values above that would count as clinically significant). Similarly, the PHOKI cut-off score is a stanine value above 7, which should be considered as clinically significant. In the present study, only adolescents without any apparent clinical psychopathology, no history of psychological/psychiatric/psychotherapeutic treatment as well as a score below the above-mentioned cut-off criteria in two questionnaires, were accepted to the control group. Four control participants with scores above average were excluded. The CON was recruited outside the clinic, as healthy study subjects without psychiatric disorders could not be recruited at our department. Subjects of both groups, CLIN as well as CON underwent the same assessment procedure with the same testing methods, carried out by the same recruiter, who had many years of professional experience.

Emotion regulation was measured by the means of the standardized self-report questionnaire FEEL-KJ *(Fragebogen zur Erhebung der Emotionsregulation bei Kindern und Jugendlichen*) [63]. It covers 15 different emotion regulation strategies (7 adaptive strategies, 5 maladaptive strategies and 3 other strategies). Adolescents rate the frequency they are using these strategies on separate five-point Likert-scales for the emotions anger, fear and sadness. The internal consistency for FEEL-KJ was between α = .69 and α = .93.

T-values were calculated using the standard values given in the manual of the FEEL-KJ [63]. They were not age or gender adjusted except for the single strategy “social support” because the manual states that neither age nor gender nor their interaction had an impact on the frequency in which the different strategies are used in children and youth.

To investigate the group differences in the use of adaptive and maladaptive strategies in general, as well as for each emotion separately, 8 t-Tests were conducted. To explore group differences in the use of single strategies, another 15 t-tests were conducted, and the level of significance was set at α = .003 (i.e., .05/15).

PHOKI [59] and CBCL/4-18 [61] are age and gender standardized surveys. The survey FEEL-KJ [63] is age and gender standardized only in the strategy “social support”.

### Statistical analysis

The statistical analysis was conducted with IBM SPSS Statistics 21.0. The raw-scores of the applied assessment instruments were converted into standard values ensuring interval scaled data. If assumptions were met, group differences were investigated using t-tests for independent samples, otherwise non-parametric tests were used.

The study was approved by the local Ethics Committee. Informed consent from all adolescents and from their parents was obtained before including them in the study.

## Results

### Demographic characteristics

In total, 66 adolescents aged 11.0 to 16.11 years were included in the study. CLIN consisted of 30 participants (14 girls, 16 boys) with an average age of 13.63 years (SD = 1.586), while CON consisted of 36 participants (25 girls, 11 boys) with an average age of 13.39 years (SD = 1.609). No significant group differences were found regarding gender (χ2 (1, N = 66) = 3.51, p = .06), the age of participants (*z* = 0.07, *p *= .500), maternal age (*z* = 1.09, *p *= .275), number of siblings [χ^2^ (2, *N* = 59) = 3.43, *p *= .180], maternal highest level of education [χ^2^ (2, *N* = 60) = 1.03, *p* = .599], or paternal highest level of education [χ^2^ (2, *N* = 55) = 4.03, *p* = .134].

There were significant group differences in paternal age (*z *= 2.57, *p* = .010), the housing situation of the family (house/flat) [χ^2^ (1, *N* = 57) = 6.37, *p* = .012], and the family status (parents living together/parents are separated) [χ^2^ (1, *N *= 60) = 7.81, *p *= .005]. More than half of CLIN members’ parents were divorced (54%), compared to just 19% of CON.

The demographics for both groups are illustrated in Table 1.

**Table 1:** Demographics of both groups CLIN and CON

Group	Gender	Age	Age_mother	Age_father	Number of siblings
CON					
N valid	36	36	35	36	36
Mean	.69	13.39	45.89	48.03	1.31
Median	1.00	13.00	44.00	47.00	1.00
Standard deviation	.467	1.609	5.930	6.729	.624
CLIN					
N valid	30	30	23	20	23
Mean	.47	13.63	47.43	51.90	1.04
Median	.00	14.00	48.00	51.00	1.00
Standard deviation	.507	1.586	5.806	6.299	.767

### Fears

Stanine-scores of the PHOKI [59] were calculated by adaptation for age and gender. The data was not normally distributed, therefore the Mann–Whitney-U-test, a non-parametric test, was used to investigate group differences. After Bonferroni-correction, the level of significance was set at α = .006 (i.e., .05/8). Cohen’s d is provided as a measure for the effect size. There were significant group differences in the total value (*z* = 3.85, *p *< .001, d = 1.06), as well as in the subscales separation anxiety (z = 6.54, p < .001, d = 2.62) and school and performance anxiety (*z* = 4.97, *p *< .001, d = 1.52), with CLIN scoring significantly higher than CON. Table 2 shows descriptive statistics of the PHOKI for both groups.

**Table 2:** Descriptive statistics of the results of the PHOKI

	Total	Dangers and death	Separation anxiety	Social anxiety	Threatening and scary	Animal phobia	Medical treatments	School and performance anxiety
CLIN								
Mean	6.23	5.27	6.07	7.87	5.97	4.97	5.73	7.27
Median	7.00***	5.00	6.00**	8.00***	6.00	6.00	6.00	8.00***
SD	2.012	1.999	2.100	1.252	2.282	2.442	2.532	1.437
CON								
Mean	4.22	4.11	4.22	4.08	5.22	5.00	5.25	4.56
Median	4.00***	4.00	4.00**	4.00***	5.00	5.00	5.00	4.50***
SD	1.570	1.720	1.742	1.763	2.085	1.836	1.538	2.063

*SD* standard deviation

** *p* < .01, *** *p* < .001

### Parents’ rating

Results of the CBCL/4-18 [61] were converted into T-values, which were adapted for age and gender. There were significant group differences regarding the total-value of the CBCL/4-18 [*t*(43.66) = 8.58, *p *< .001, d = 2.30], with CLIN scoring higher than CON. Both groups also differed significantly in both the subscales internalizing problems [*t*(41.86) = 9.74, *p *< .001, d = 2.63], and externalizing problems [*t*(41.74) = 2.03, *p *= .049, d = 0.54], with CLIN scoring higher than CON. Table 3 contains means and standard deviations for both groups.

**Table 3:** Descriptive statistics of the CBCL/4-18

CBCL/4-18-scales	N	Mean	SD
Internalizing problems			
CON	36	45.69***	6.944
CLIN	27	68.74***	10.719
Externalizing problems			
CON	36	44.92*	8.230
CLIN	27	50.63*	12.759
Total			
CON	36	44.64***	7.235
CLIN	27	64.85***	10.513

Means and standard deviations of the CBCL/4-18 for both groups (CLIN and CON)

* *p* < .05, *** *p* < .001

### Emotion regulation and SAD

In the test construction of the FEEL-KJ no gender differences were found except for the strategy “social support,” therefore no gender or age adjusted standardized values are provided in the manual. Accordingly, we did not find any gender differences in the use of emotion regulation strategies.

### Adaptive emotion regulation

**Fig. 1:**
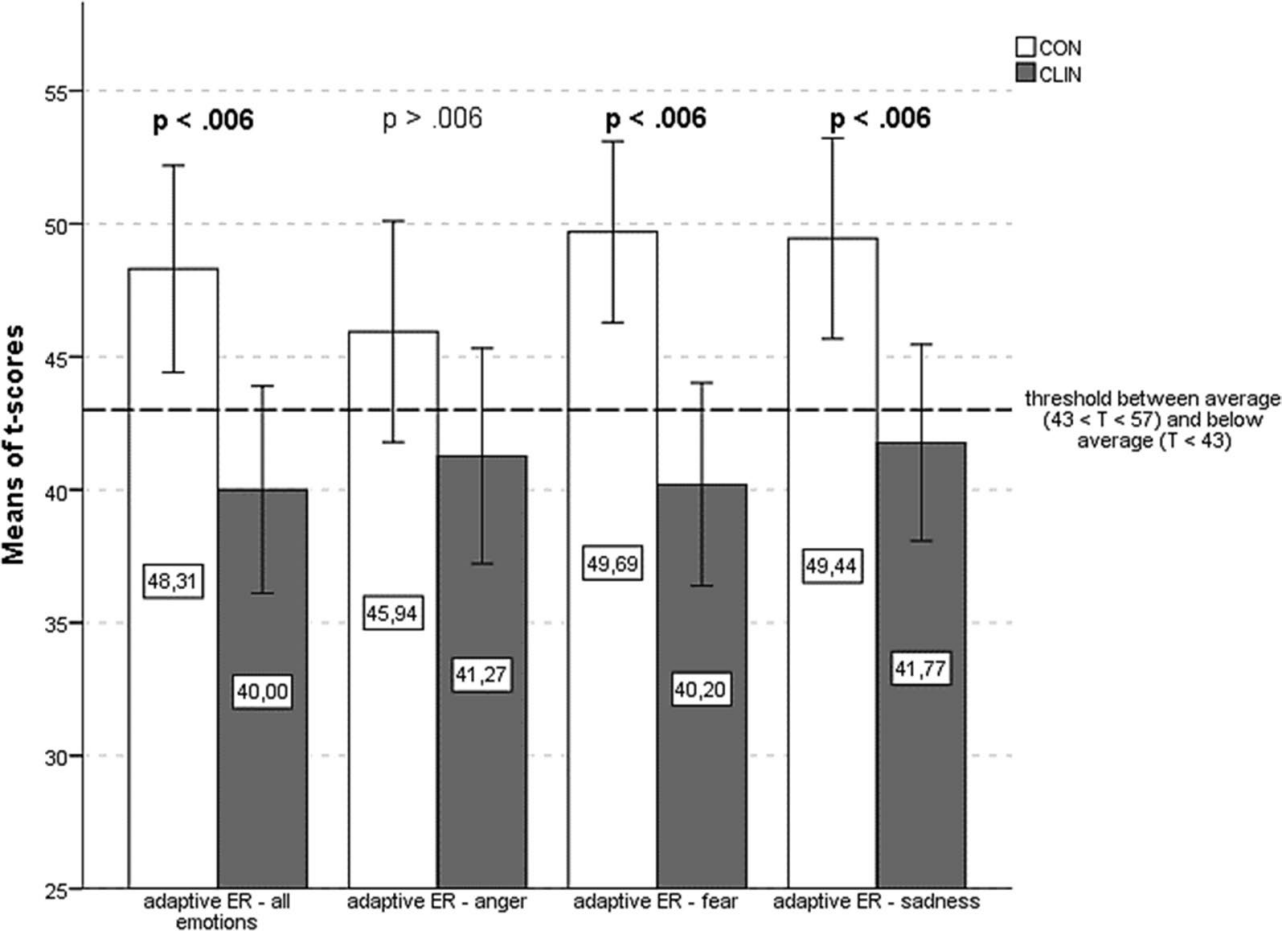
Adaptive ER. Means of adaptive ER over all emotions and for each emotion (anger, fear, sadness) separately for both groups (CLIN and CON) with error bars marking the 95% CI. Applying Bonferroni-correction the level of significance was set at α = .006. Significant differences are highlighted. The threshold between the average range and the below-average range is marked by a horizontal line at T = 43

Summed up over all three examined emotions (*anger, fear, sadness*), there was a significant difference between CLIN (*M *= 40.00, *SD *= 10.42) and CON (*M *= 48.31, *SD *= 11.47) in the frequency of using adaptive strategies [*t*(64) = 3.05, *p *= .003]. CLIN youth used adaptive ER strategies significantly less often than CON. The effect size was estimated with Cohen’s d, d = .75. Additionally, CLIN showed lower scores in the use of adaptive ER strategies in the context of *fear* [*t*(64) = 3.79, *p *< .001, d = 0.93] and *sadness* [*t*(64) = 2.93, *p *= .005, d = 0.72]. No significant difference was found in the use of adaptive ER strategies in the context of *anger* [*t*(64) = 1.62, *p *= .109]. Figure 1 illustrates the group differences in the use of adaptive ER strategies.

### Maladaptive emotion regulation

**Fig. 2:**
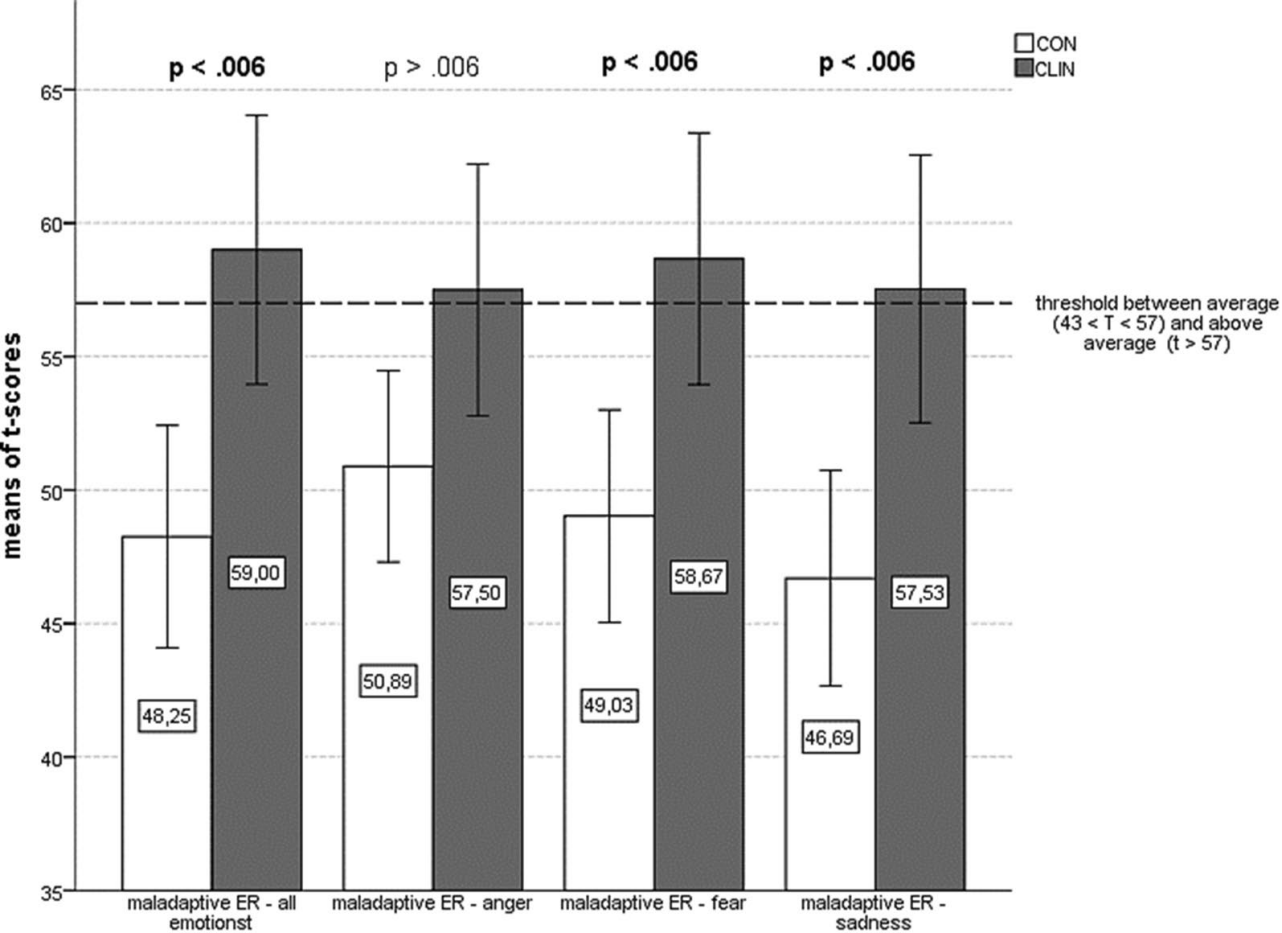
Group differences in the use of maladaptive ER strategies. There was a significant difference in the use of maladaptive strategies over all three emotions between CLIN (M = 59.00, SD = 13.48) and CON (M = 48.25, SD = 12.33) [t(64) = 3.38, p = .001, d = 0.84], with CLIN reporting significantly more use of maladaptive ER strategies than CON

There was a significant difference in the use of maladaptive strategies over all three emotions between CLIN (*M *= 59.00, *SD* = 13.48) and CON (*M *= 48.25, *SD *= 12.33) [*t*(64) = 3.38, *p *= .001, d = 0.84], with CLIN reporting significantly more use of maladaptive ER strategies than CON. Examining the results for the three emotions separately, there were significant group differences regarding the emotion *fear* [*t*(64) = 3.21, *p *= .002, d = 0.79] and *sadness* [*t*(64) = 3.496, *p *= .001, d = 0.62], with CLIN scoring higher in both cases. Applying Bonferroni-correction the level of significance was set at α = .006. The group difference regarding the emotion *anger* failed to reach significance [*t*(64) = 2.31, *p *= .024]. Figure 2 illustrates the group differences in the use of maladaptive ER strategies.

### Single emotion regulation strategies

Examining group differences on the basis of single ER strategy use, only one *t* test comparison reached significance after Bonferroni-correction. CLIN (*M *= 43.23, *SD* = 9.17) and CON (*M *= 51.28, *SD* = 10.65) differed significantly in the use of the adaptive ER strategy *reappraisal* [*t*(64) = 3.25, *p* = .002, d = 0.81], with CLIN reporting less use of *reappraisal* than CON. There tended to be group differences regarding the maladaptive ER strategies *withdrawal* [*t*(64) = 2.84, *p* = .006, d = 0.70] and *rumination* [*t*(64) = 2.67, *p* = .01, d = 0.66], as well as the adaptive ER strategy *problem*-*solving* [*t*(64) = 2.71, *p* = .009, d = 0.68], with CLIN reporting more use of *withdrawal* and *rumination* and less use of *problem*-*solving* than CON. Table 4 shows means, standard deviations, and t-test comparisons of the 4 ER strategies mentioned above for both groups.

**Table 4:** Means and standard deviations of ER strategies for both groups

	CLIN		CON	
	M	SD	M	SD
Reappraisal	43.23**	9.17	51.28**	10.65
Withdrawal	60.33	12.16	52.42	10.47
Rumination	53.17	9.65	46.53	10.41
Problem-solving	42.33	10.78	49.47	10.54

** *p* < .01

A stepwise binary logistic regression was performed to explore if the use of certain single ER strategies could predict group membership. All of the seven adaptive and five maladaptive ER strategies were thereby included. Table 5 illustrates the three steps of the regression and the final regression model. No outliers and no influential cases were detected; therefore, all cases were included. The final regression model found three ER strategies to be predictors: *reappraisal, rumination,* and *withdrawal*. In other words, the frequency of the use of the ER strategies *reappraisal*, *rumination,* and *withdrawal* significantly predicted the membership of participants to either CLIN or CON. The exp *b*-value showed that as the use of *reappraisal* increased, while keeping rumination and withdrawal constant, the probability of belonging to CLIN decreased. In contrast, as the use of rumination or withdrawal increased, the probability of belonging to CLIN increased. There was no collinearity between the predictors influencing the accuracy of the model. In total, the model with three predictors could correctly assign 75.8% of the participants to either CLIN or CON. R^2^ was .42, so the three predictors explained 42% of the variance of group membership (Table 6).

**Table 5:** Stepwise binary logistic regression-model of single ER strategies

	B	Standard error	Wald	Exp *b*	95% CI for exp *b*	
					Lower	Upper
Step 1						
Reappraisal	− .084**	.030	8.134	.919	.867	.974
Constant	3.781	1.397	7.320	43.859		
Step 2						
Reappraisal	− .108**	.034	9.982	.897	.839	.960
Rumination	.101**	.036	7.755	1.107	1.030	1.188
Constant	− .057	1.795	0.001	.945		
Step 3						
Reappraisal	− .108**	.035	9.278	.898	.837	.962
Withdrawal	.058*	.029	4.118	1.060	1.002	1.121
Rumination	.092*	.036	6.491	1.096	1.021	1.177
Constant	− 2.922	2.318	1.589	.054		

Anmerkung: R^2^ = .42 (Nagelkerke) after step 3; Model χ^2^ (3) = 24.97, p < .001

* *p* < .05, ** *p* < .01

**Table 6:** Partial correlation between social anxiety and ER

	Adaptive ER	Maladaptive ER
PHOKI social anxiety		
Correlation	.151	.530
Significance (two-tailed)	.230	.000
Degrees of freedom	63	63

Partial correlation between the subscale *social anxiety* of the PHOKI and the use of adaptive and maladaptive ER strategies respectively controlling for group membership

### Association and relation between social anxiety disorder and the use of emotion regulation

CLIN youth used adaptive ER strategies significantly less often than CON. Examining emotions (*anger, fear, sadness*), there was a significant difference between CLIN (*M *= 40.00, *SD *= 10.42) and CON (*M *= 48.31, *SD *= 11.47) in the frequency of using adaptive strategies [*t*(64) = 3.05, *p *= .003].

There was a significant difference in the use of maladaptive strategies over all three emotions between CLIN (*M *= 59.00, *SD* = 13.48) and CON (*M *= 48.25, *SD *= 12.33) [*t*(64) = 3.38, *p *= .001, d = 0.84], with CLIN reporting significantly more use of maladaptive ER strategies than CON.

### Regarding single emotion regulation strategies

CLIN (*M *= 43.23, *SD* = 9.17) and CON (*M *= 51.28, *SD* = 10.65) differed significantly in the use of the adaptive ER strategy *reappraisal* [*t*(64) = 3.25, *p* = .002, d = 0.81], with CLIN reporting less use of *reappraisal* than CON. Regarding the maladaptive ER strategies within the CLIN and CON *withdrawal* [*t*(64) = 2.84, *p* = .006, d = 0.70] and *rumination* [*t*(64) = 2.67, *p* = .01, d = 0.66], as well as the adaptive ER strategy *problem*-*solving* [*t*(64) = 2.71, *p* = .009, d = 0.68], with CLIN reporting more use of *withdrawal* and *rumination* and less use of *problem*-*solving* than CON.

## Discussion and interpretation

The aim of this study was to investigate the ER of adolescents with a diagnosis of SAD.

The results of Sung [64] indicate that individuals with SAD consider their ability to successfully regulate their emotions to be lower than that of healthy controls. In addition they found that a strong belief in one’s emotion regulation skills is associated with a higher quality of life. Results of the present study demonstrated significant differences in the use of adaptive and maladaptive ER strategies between socially anxious adolescents and a healthy control group, with CLIN youth scoring significantly lower in adaptive ER strategy use and significantly higher in maladaptive ER strategy use than CON youth. While this was true regarding all examined emotions (*anger, fear, sadness*) together, as well as for fear and sadness separately, there was no significant group difference in the use of adaptive and maladaptive ER strategies in the context of *anger*.

Our results are partly in line with the study of Schäfer et al., which used a meta-analysis of 35 studies in adolescents (aged 13–18 years) to confirm that healthy individuals engaged more in *reappraisal, problem*-*solving (adaptive strategies)* and showed less avoidance, suppression and rumination (maladaptive strategies) when compared to individuals with anxiety [41].

Based on the results of the present study, adolescents with SAD should get to know the use of adaptive emotion regulation strategies such as reappraisal and *problem*-*solving* ideally at the beginning of the therapeutic process; as the gradual acquisition of positive emotion regulation strategies significantly improves the self-esteem of adolescents and increases their motivation for further therapeutic interventions.

Earlier studies, as well as one including a meta-analysis [65], have already reported associations between maladaptive ER and anxiety disorders [57, 66, 67]. Our findings are in line with these former studies. The literature is inconsistent regarding adaptive ER. While our results are in line with those of Lange and Tröster [57], which too found that children and adolescents with SAD use adaptive ER strategies less often than healthy controls, there are studies with contradictory findings. Whereas the above mentioned meta-analysis by [65] found a significant negative association between adaptive ER strategies and anxiety disorders for only one of the examined strategies, namely *problem*-*solving*. In the study of [67], children and adolescents with SAD used not only maladaptive ER strategies more often than a healthy control group, but also some adaptive ones (*refocus on planning*, *acceptance*). Tan et al. did not find any differences in the use of adaptive or maladaptive ER strategies between children and adolescents with anxiety disorders and healthy controls [15]. However, important strategies like *reappraisal* and *problem*-*solving* were not included in this study. On top of that, it did not include how participants dealt with the emotion *fear*, which is important in the context of anxiety disorders.

Despite the group differences in the use of both adaptive and maladaptive ER strategies, when controlling for group membership we found a significant positive correlation between maladaptive ER and social anxiety in adolescents. We did not find a significant association between adaptive ER and social anxiety. Therefore, the increased use of maladaptive ER strategies seems more prominent than the decreased use of adaptive strategies. This result is in line with Aldao [65], who reported only small non-significant correlations between adaptive ER and anxiety disorders. In a subsequent study, they showed that a flexible implementation of adaptive strategies dependent on the situational context was negatively associated with psychopathology, and not the mere frequency of the adaptive ER strategy use [68].

Among all the examined ER strategies, we found *reappraisal*, *rumination,* and *withdrawal* to be significant predictors of membership to either the clinical or the control group. An increased use of *rumination* has been reported to be associated with SAD [57, 67], which supports the present finding. Additionally *rumination* has been found to have a more negative influence on children with SAD compared with healthy controls [15]. Other than Lange and Tröster’s [57] finding that children with SAD use the strategy *withdrawal* significantly more often, there are no additional studies on the association between *withdrawal* and SAD. However, the construct *withdrawal,* as assessed by the FEEL-KJ, shares qualities with the strategy *suppression,* which is not directly assessed by the FEEL-KJ. Both strategies focus on keeping one’s emotions to oneself. The negative consequences of *suppression,* [50] as well as its association with SAD, are well documented [68, 69]. Given the similarities between *withdrawal* and *suppression,* our finding is in line with previous research. According to the cognitive model of SAD by Clark and Wells [11], individuals with SAD believe evaluation by others to be ruthless and therefore fear rejection if they show negative emotions, which may explain the finding that those with SAD prefer to use *suppression*. If confirmed, the result that only the increased use of maladaptive ER is associated with social anxiety may have other implications for the psychotherapy of SAD.

Based on the knowledge that negative emotion regulation strategies in adolescents with SAD play an important role in the development and maintenance of their psychopathology, the adequate handling of negative emotion states should be used as a central element of the therapeutic process at the beginning of psychotherapy (CBT).

Based on the results of the present study, adolescents with SAD should get to know the use of adaptive emotion regulation strategies such as reappraisal and *problem*-*solving* ideally at the beginning of the therapy process. To increase and maintain motivation for further therapeutic interventions it is important to improve self-esteem in adolescents by gradual acquisition of positive emotion regulation strategies.

## Conclusions

The main finding of this study was a significant positive correlation between maladaptive ER and social anxiety disorder in adolescents. There is a strong medical recommendation to include the reduction of maladaptive ER strategies from the very beginning of the psychotherapy process. When evaluating single ER strategies, the current study found CLIN reporting less use of *reappraisal* than CON.

Adolescents with SAD used the strategy *reappraisal* significantly less often than healthy controls. This finding is supported by several studies reporting negative associations between *reappraisal* and anxiety disorders [14, 70, 71]. These findings provide a rationale for special therapy programs concentrating on the establishment of different adaptive ER strategies (including *reappraisal*) in patients with different mental health problems [72, 73].

Regarding the maladaptive ER strategies within the CLIN and CON *withdrawal* and *rumination*, as well as the adaptive ER strategy *problem*-*solving*, CLIN reported more use of *withdrawal* and *rumination* and less use of *problem*-*solving* than CON. In line with the study of Schäfer et al. [41] rumination and its treatment has a wide effect on the outcome of psychopathology in adolescents with anxiety symptoms. Also in line with the study of Schäfer et al. [41] problem solving is related to a lower level of anxiety symptoms when coping with demanding emotional events.

To our knowledge there is little known about SAD in adolescents and ER and specific psychotherapeutic interventions in combination with emotion regulation strategies. Further studies should aim to understand the role of emotion regulation strategies in the treatment of SAD in adolescence to improve the treatment outcome.

## Limitations

The current study has some limitations. First, comorbidities were not assessed and therefore not controlled for. Epidemiologic studies show that SAD patients often suffer from additional internalizing disorders, which could have influenced our results. Second, the investigation of ER strategy use is based on self-reports of the participating adolescents. In addition, sample size is rather small and no gender-matching was done which could affect generalizability.

Further studies with a larger and comprehensive sample should reevaluate the ER results with appropriate gender-matching, which also considers the comorbid disorders and compares them with these results.

In this sense, the long-term psychotherapy for affected young people with SAD can be adapted gradually with appropriate adaptive and maladaptive emotion regulation strategies in order to optimized treatment for long-term outcome.

## Strengths

One of the strengths of this study was the inclusion of a clinical group with a primary diagnosis of SAD confirmed by a mental health professional. There have only been a few studies that included clinical groups, particularly with children and adolescents. In the meta-analysis by Aldao et al. for example, there was no study that involved a clinical group of children and adolescents [65]. In addition, the current study investigated ER in the context of three distinct emotions (anger, fear, and sadness) and examined 15 different ER strategies, which provides a comprehensive insight into the specific characteristics of ER in adolescents with SAD.

## Future directions

Future studies are needed to investigate the causal associations between the use of maladaptive ER strategies and SAD in adolescents. In addition, further research is needed regarding the association of adaptive ER strategy use and SAD in order to address the inconsistency in todays literature. To our best understanding there is little knowledge about the SAD in adolescents and ER as well as specific psychotherapeutic interventions in combination with emotion regulation strategies. Therefore, further studies should aim to understand the role of emotion regulation strategies in the treatment of SAD in adolescence. Incorporating more ER components into psychotherapeutic treatment could increase treatment efficacy [74].

Such research could improve the methods of screening and psychotherapy in addition to enhancing the efficacy of current treatment protocols.

## Data Availability

All data and material are available at the Department of Child and Adolescent Psychiatry at the Medical University Vienna.

## Abbreviations

SAD: social anxiety disorder
ER: emotion regulation
CLIN: clinical group
CON: control group
CBT: cognitive behavior therapy
